# Progress in Surgery

**Published:** 1900-07-28

**Authors:** 


### Progress in Surgery.

### HERNIA.

**Hernia of the Bladder.**—This subject has been very thoroughly discussed by B. G. A. Moynihan[1] in the the third Arris and Gale Lecture, of which we give an abstract. During the last decade the number of recorded cases has been steadily increasing, until now the number approximately reaches 200. All the earlier cases contained a large amount of the bladder; some contained the whole bladder, and two at least contained the prostate also. Probably the fact that the inguinal canal is now so frequently slit up in the radical cure of hernia accounts for the increasing frequency with which hernias are found to contain small portions of the bladder. The percentage occurrence of cystocele in hernias was about 1 per cent. in 2,543 cases of hernia, all submitted to operation without selection. The rarity of recorded examples seems to be due rather to a want of careful examination than to an infrequent existence of the cases. Four cases only have been recorded in children, and it may be broadly affirmed that hernia of the bladder is a disease of the aged and enfeebled. The majority occur between the ages of 50 and 60. There are three distinct forms of cystocele: (1) The intra-peritoneal, (2) the para-peritoneal, and (3) the extra-peritoneal. In the intra-peritoneal variety there is a complete hernial sac in the position of an oblique inguinal hernia. Into this a portion of the bladder, completely covered with peritoneum, descends. It is the upper part of the posterior surface of the bladder that is implicated. Moynihan only found four inguinal cases and one femoral of this variety of hernia of the bladder in 200 cases. In the para peritoneal form there is a sac of the oblique or direct variety, on the inner side of which lies the bladder in such a manner that the inner wall of the sac is the serous covering of the outer wall of the bladder. The sac may be large and capacious and a mere fringe of bladder be present in the canal, or the bladder involved may be large and bulky, and the peritoneal descent trivial. In about half of the total cases the bladder has been thickly covered with fat. The extra-peritoneal form of cystocele is a rare form. It seems open to question whether any case of this form should be definitely accepted without the evidence of a careful dissection, for a small co-existent hernial sac may be easily missed. These hernias are usually very small, and are always of the direct variety. The bladder wall in hernia of the bladder is more frequently thinned than thickened, and it may appear as thin as serous membrane. The extruded portion of the viscus may have a phosphatic calculus developed in it, and has been known to produce an abscess in the groin, The bladder may be pulled down into the inguinal canal by traction during operations for the cure of hernia. These cases are described by Hermes as "operative cystoceles." The thin, flaccid bladder, met with in some cases of enlarged prostate, stricture, and cystitis, is more likely to be herniated than a normal bladder. It is not a diverticulum of the bladder in the true sense, which presents in these herniæ, though the communication between the herniated and the other part of the bladder may be very narrow. The fat surrounding the bladder, as mentioned above, may be ætiologically related to these herniæ, either by fixing or drawing the bladder at or near to the internal abdominal ring. Other causative factors are permanent vesical distension, motor insufficiency of the bladder, laxity of the abdominal walls with undue potency of the inguinal canal and rings, and increase of intra-abdominal pressure. Not more than 5 per cent. of the cases have been diagnosed before operation. The signs, when present, are of hernia which at times contains fluid which can be reduced by pressure. The size of the hernia is subject to rapid fluctuation. Usually the swelling is in part irreducible. The swelling is not translucent. The irreducible part is doughy and thick, being made up of the "lipome herniaire." The chief symptom is "miction en deux temps," associated with a lessening of the hernial swelling. The second flow of urine is caused or assisted by pressure in the hernia. In some cases a catheter has been passed into the bladder and out by the inguinal canal, so that the beak could be felt in the hernia. There may be symptoms such as vomiting, distension, and obstruction of fæces and flatus, suggesting strangulation, but never so acute. The treatment is by operation. This will enable the patient to wear a truss, which is otherwise impossible. If accidentally wounded the bladder must be sutured, the sutures not penetrating the mucous membrane.

**Volvulus in Association with Hernia.**—R. Lawford Knaggs[2] has contributed a paper on this subject, suggested by two cases in his own practice, in which strangulation was dependent on a volvulus. The cases collected are arranged in the following four groups: (1) Volvulus of the hernial contents, the neck of the volvulus being within the sac or close to the hernial aperture; the volvulus may affect all the intestinal contents of the sac, or only a portion. (2) Volvulus in which the hernial contents are implicated, but where the neck and some of the affected coils lie within the abdomen. (3) Volvulus within the abdomen produced by the reduction of the hernia. (4) Volvulus within the abdomen from some predisposing condition, more or less directly connected with a hernia. Of the first variety, Knaggs quotes seven recorded cases, two of these being of duodenal hernia. The bowel may be forced in the twisted state into the sac, or the twist may occur or be produced in the sac by irregular peristalsis. Cases of volvulus in duodenal hernia form a class by themselves. The volvulus may exist for some time without giving rise to obstruction or strangulation. Traction is considered a factor of great importance in the production of the twist. A coil of intestine in the abdomen may be in the reversed position without being in a condition of volvulus, but if it is suddenly forced into and retained in the sac of a hernia the two ends of the coil are put on the stretch, and the tension approximates them and further develops the twist. The stretching also narrows the lumen, and favours obstruction and strangulation. Volvulus seems to be rather common in duodenal hernia, probably as a consequence of excited intestinal movements, but strangulation is rare. Of cases in which the neck of the volvulus lay within the abdomen, Knaggs gives records of five; two of these occurred in his own practice. They usually run a very urgent and rapid course, and a long length of bowel is often involved, averaging a couple of yards. The key to the solution of the case lies within the abdomen, and an extended incision is usually necessary to reach it. The mechanism of obstruction is as follows. A twist of the bowel occurs within the abdomen, and a portion of the twisted loop becomes engaged in the hernial sac. The twist cannot then right itself. More bowel escapes until one limb of the twist becomes taut. The stretched segment lies entirely within the abdomen, its peripheral end being at the neck of the sac. Thus the stretched limb is converted into a stout band, and under it the other limb and a varying portion of mesentery are compressed, and often strangled. In one of Knaggs's cases, however, the strangulation occurred through one limb hanging over the other, which was more like a slack wire than a tight band. Thus these cases have the mixed features of strangulation by volvulus and of strangulation either over or under a band. This explains the unequal degrees of engorgement sometimes exhibited by the two ends of the volvulus. The portion of the bowel near and passing into the part acting like a band may be hardly at all engorged, whilst the other end passing to the compressed end may be engorged to a marked degree. Tenderness of the abdomen may be an important sign of this condition. It may be localised over the neck of the twist, and the hernia itself may be free from tenderness, and thus a strong suspicion may be raised that the cause of the trouble is intra-abdominal. Knaggs points out that it is no use merely reducing these hernias by operation, which will leave in the abdomen a couple of yards or so of blood-sodden infiltrated and inflamed intestine, teeming with micro-organisms producing toxins sure to be fatal. Irrigation and washing away the fetid contents, as Kocher advocates in intestinal obstruction, does not remove the organisms which have already invaded the intestinal wall. The only successful case Knaggs draws attention to is one in which Dreesmann removed the unhealthy intestine, which was 2·15 metres in length, and he thinks such resection to be the treatment strongly indicated for these cases, even though the bowel is not in a state of actual gangrene. Probably the amount of shock caused by removing a few feet of bowel is not greater than that caused by removing a few inches only. Knaggs describes two cases of the third group, that in which the reduction of the hernia produces a volvulus. In one of these the reduction had forced a hernia of the cæcum into the left hypochondrium, leaving a kink which produced obstruction at its junction with the ascending colon. In such cases this persistence of symptoms would lead to the inference, most probably, that reduction *en masse* had occurred.

[1] Lancet, March, 1900. [2] Annals of Surgery, April, 1900.

